# Epidermal growth factor induces HCCR expression via PI3K/Akt/mTOR signaling in PANC-1 pancreatic cancer cells

**DOI:** 10.1186/1471-2407-10-161

**Published:** 2010-04-27

**Authors:** Zekuan Xu, Yi Zhang, Jiakai Jiang, Yang Yang, Ruihua Shi, Bo Hao, Zhihong Zhang, Zuhu Huang, Jin W Kim, Guoxin Zhang

**Affiliations:** 1 Department of General Surgery, the First Affiliated Hospital of Nanjing Medical University, Nanjing 210029, China; 2 Department of Gastroenterology, the First Affiliated Hospital of Nanjing Medical University, Nanjing 210029, China; 3 Department of Pathology, the First Affiliated Hospital of Nanjing Medical University, Nanjing 210029, China; 4 Department of Infectious Diseases, the First Affiliated Hospital of Nanjing Medical University, Nanjing 210029, China; 5 Department of Emergency Surgery, the First Affiliated Hospital of Nanjing University of Chinese Medicine, Nanjing 210029, China; 6 Department of Obstetrics & Gynecology, college of Medicine, The Catholic University of Korea, Seoul, 137-040, Korea

## Abstract

**Background:**

Human cervical cancer oncoprotein 1 (HCCR-1), reported as a negative regulator of p53, is over-expressed in a variety of human cancers. However, it is yet unknown whether HCCR-1 plays any role in pancreatic cancer development. The aim of this study was to investigate the effect of epidermal growth factor on the expression of HCCR in pancreatic cancer cells, and to explore if PI3K/Akt/mTOR signaling pathway mediated this expression.

**Methods:**

A polyclonal antibody against HCCR protein was raised by immunizing Balb/c mice with the purified recombinant protein pMBPc-HCCR. Tissue samples were constructed on a tissue chip, and the expression of HCCR was investigated by immunohistochemistry assay and Western blotting. Pancreatic cell line, PANC-1 cells were stably transfected with plasmids containing sense-HCCR-1 fragment and HCCR siRNA fragment. MTT and transwell assay were used to investigate the proliferation and invasion of stable tansfectants. The specific inhibitor of PI3K and mTOR was used to see if PI3K/mTOR signal transduction was involved in the induction of HCCR gene expression. A Luciferase assay was used to see if Akt can enhance the HCCR promoter activity.

**Results:**

HCCR was up-regulated in pancreatic tumor tissues (mean Allred score 4.51 ± 1.549 *vs*. 2.87 ± 2.193, P < 0.01), especially with high expression in poorly differentiated pancreatic cancer. The growth of cells decreased in HCCR-1 siRNA transfected cells compared with vector transfectants. The number of invasion cells was significantly lower in HCCR-1 siRNA transfected cells (24.4 ± 9.9) than that in vector transfectants (49.1 ± 15.4). Treatment of PANC-1 cells with epidermal growth factor increased HCCR protein level in a dose- and time-dependent manner. However, application of LY294002 and rapamycin caused a dramatic reduction of epidermal growth factor-induced HCCR expression. Over-expression of exogenous constitutively active Akt increased the HCCR promoter activity; in contrast, dominant negative Akt decreased the promoter activity.

**Conclusions:**

EGF-induced HCCR-1 over-expression is mediated by PI3K/AKT/mTOR signaling which plays a pivotal role in pancreatic tumor progression, suggesting that HCCR-1 could be a potential target for cancer therapeutics.

## Background

Pancreatic cancer is one of most common malignant tumors with poor prognosis, and its incidence is on the rise globally. The five-year survival rate is less than 5 percent among pancreatic cancer patients with rare complete remission [1-5]. Although a large number of potential proteins and gene-based markers have been used for diagnosis of pancreatic cancer, the established marker so far is CA19-9 with better diagnostic sensitivity and specificity of 68% and 76%, respectively [6-8].

Recent molecular investigations have elucidated complex genetic mechanisms of cancer that especially involve multiple signal transduction pathways. These findings enable us to develop molecular medicines targeting specific genetic molecules in the pathways. Cancer is a genetic disease; i.e., dysfunctions of multiple genes including active oncogenes and inactive tumor suppressor genes play crucial roles in the development and progression of the disease. Many of these dysfunctioning molecules comprise signaling pathways, which indicates that cancer is a signaling disorder. Aberrantly activated signal transduction systems are vital for the sustenance of cancer, which is often compared to a state of "addiction". This extent of dependence upon aberrant signaling systems in cancer implies that shutting down the signaling would cause the cancer to vanish.

The PI3K-Akt pathway is major signaling pathway involved in the oncogenesis of many types of cancers [9]. PI3K is a heterodimer of the 85-kDa and 110-kDa subunits and has a tyrosine kinase activity. PI3K mediates an activating signal from the growth factor receptors to Akt, which is a kinase that translocates into the nucleus and phosphorylates a variety of target molecules to mediate signals, including mTOR. mTOR is a serine/threonine kinase implicated in the regulation of translation initiation [10]. The function of mTOR is associated with the PI3K-Akt pathway via TSC [11]. Although no mutations in PI3K or Akt1 have been reported so far, evidence suggests that the PI3K/Akt pathway is active in pancreatic cancers [12-14], which indicates that the pathway is a putative therapeutic target in such cancers.

Human cervical cancer oncogene (HCCR) was firstly identified in primary cervical cancers and cervical cancer cell lines by using differential display RT-PCR approach [15-17]. The HCCR gene is classified into two isoforms, wild type HCCR-1 which encodes 360 amino acids (42 KD) and its alternative splicing variant, HCCR-2 which encodes 304 amino acids (36 KD) [15]. Previous study suggested that nude mice injected with NIH/3T3 cells stably transfected with HCCR formed tumors within 4 weeks. NIH/3T3 cells stably transfected with HCCR fragment showed increased transformation efficiency and more colony formation in soft agar, and it is also found that HCCR involves in p53 stabilization, decreased expressions of p53-responsive gene such as p21 and Bax, suggesting that HCCR may function as a negative regulator of p53 [15,16]. HCCR was also validated as a biomarker for both human hepatocellular carcinoma and breast cancer [18,19]. HCCR-1 and DP1 which play a tumor-suppressor role in colorectal cancer were supposed to regulate each other negatively by interaction [20].

To determine the regulatory pathway involved in the HCCR-1 gene expression, Cho GW et al searched the 5-flanking region of HCCR-1 and found that the HCCR-1 oncogene expression is regulated by the PI3K/Akt signaling pathway in K562, MCF-7 and A549 cells [21]. HCCR-1 is not only over-expressed in cervical cancer tissues, but also in several other cancers including leukemia, lymphoma, and carcinomas of breast, kidney, ovary, stomach, and colon [15]. Despite of this, little is known about the role of HCCR-1 in pancreatic cancer development. Here in this study, we demonstrate that HCCR-1 is responsible for pancreatic cancer via EGF mediated-PI3K/Akt/mTOR signaling pathway.

## Methods

### Preparation of HCCR polyclonal antibody

The cDNA encoding the C-terminus of HCCR (from the 167th to 360th amino acid residues) was cloned into the pMBP-c containing tags of MBP and polyHis (Fermentas MBI) vector. The construct was then transformed into the E. coli Top10F' (Fermentas MBI). Expression of HCCR C-terminal polypeptide was induced by IPTG (0.4 mM/L). The recombinant product was purified by nick-nitrilotriacetic acid (Ni-NTA)-affinity chromatography. BALB/c mice were immunized by intrasplenic deposition of 1 μg of the purified fusion protein attached to PVDF membrane for the first time [22]. Two weeks later, mice were immunized again by intra-peritoneal injection with 50 μg of polypeptide mixed with Freund's complete adjuvant. The polyclonal anti-HCCR serum was tested for its efficiency and specificity by indirect ELISA and Western blot.

### Sample selection and tissue chip construction

178 cases of pancreatic tumor, 47 cases of paraneoplastic tissue and benign tumor were obtained from sample library of Shanghai Biochip Corporation, including 159 cases of adenocarcinoma, 7 cases of adenosquamous carcinoma, 8 cases of mucoid adenocarcinoma, 2 cases of carcinoid, 1 case of spindle cell malignant tumor and 1 case of acinic cell carcinoma, 36 cases of paraneoplastic tissues and 11 cases of pancreatic benign tumors. The age of the patients ranged from 30 years to 86 years. Of the patients with pancreatic carcinoma, 111 were males, and 67 patients were females. Pathologic diagnosis was proved by two experienced pathologists from two different hospitals. 99 cases accompany nerve infiltration and 36 cases with lymph node metastasis were determined. Tissue chip was constructed by Shanghai Biochip Corporation.

### Cell Culture and Chemical Compounds

Human cell lines from ATCC, were maintained in Dulbecco's minimal essential medium(Gibco, Grand Island, NY) supplemented with 10% fetal bovine serum, 100 units/mL penicillin, and 100 μg/mL streptomycin at 37°C in a 5% CO_2_-humidified atmosphere. Cells were plated at 5 × 10^5 ^per well in 6-well plates. For growth factor deprivation, the medium was made without serum, EGF, and insulin. Cells were grown to 60% to 70% confluency, then starved in serum-free DMEM for 24 hours, then the cells were pretreated with inhibitors for 1 hours incubated in the presence of EGF for 24 hours and extracted and subjected to Western blot analysis. The human recombination protein EGF was purchased from Peprotech. LY294002 were purchased from Cell Signaling Technology. Rapamycin were obtained from Sigma.

### DNA constructs and transfection

The constructs of Akt kinase, constitutively active Akt kinase, and dominant-negative Akt kinase (K179M) in the pCMV-6 vector or in the retrovirus vector pLNCX were generously provided by Thomas Franke [23,24]. The constitutively active Akt kinase and dominant-negative Akt kinase were re-cloned into pcDNA3.1 vector in our laboratory. The construct of HCCR-1 in pcDNA3.1 was kindly gifted from Dr. Jin Woo Kim [21] (The Catholic University of Korea). HCCR-1 siRNA were constructed in pGCsi-H1/Hygro/NEGative vector by GeneChem company, Shanghai, China. The sequences of the selected region to be targeted by siRNA for HCCR were: SR54-3F: TGCTGAATCACATCGGAATGCTCATTGTTTTGGCCACTGACTGACAATGAGCACCGATGTGATT; and SR54-3R: CCTGAATCACATCGGTGCTCATTGTCAGTCAGTGGCCAAAACAATGAGCATTCCGATGTGATTC. PANC-1 cells in exponential growth were seeded into 6-well plates at a concentration of 1 × 10^5^/ml. After 24 hours, cells were transfected with 2 μg of DNAs of constitutively active Akt (CA-Akt), dominant negative Akt(DN-Akt), HCCR-1 siRNA and HCCR-1-pcDNA3.1 by lipofectmine 2000 (Invitrogen, Carlsbad, CA), respectively. Culture medium was replaced after 6 hours of incubation, and medium containing 500 μg/mL G418 was used for screening 48 hours later. About 3 weeks later, ten G418-resistant clones were selected with a cloning ring for amplification in culture.

### Immunohistochemistry

The immunostaining was performed manually at room temperature by using the UltraSensitive SP immunohistochemistry kit (Maixin Biotech, Fuzhou). PBS replaced the murine polyclonal anti-HCCR serum (1:100) as a negative control. Using the Allred 8-unit system, we determined the tumor epithelial cells proportion score and intensity score. The stain was examined by 2 independent pathologists using the Allred 8-unit system with the combination of a proportion score from 0 to 5 and an intensity score from 0 to 3. The proportion score included the fraction of positively stained tumor cells and was as follows: 0 = none, 1 = <1/100th; 2 = 1/100th to 1/10th; 3 = 1/10th to 1/3; 4 = 1/3 to 2/3; 5 = >2/3. The staining intensity score was as follows: 0 = none; 1 = weak; 2 = intermediate; 3 = strong [25,26].

### Western Blot Analysis

For Western blot analysis, tissues and cells were lysed by lysis buffer, and the lysates were collected. The protein were diluted in the sample buffer(250 mM Tris-HCl, 4% SDS, 10% glycerol, 0.006% bromophenol blue and 2% β-mercaptoethanol) and boiled for 5 min after measured the concentration with the BCA protein assay. Equivalent volumes of lysates containing 20 ug of total protein were loaded and size-fractionated using 10% SDS-polyacrylamide gels. Proteins were transferred onto nitrocellulose membrans at 100 V for 90 min. Subsequently, membranes were incubated with 1:500 dilution of murine polyclonal anti-HCCR-1 antibody (Tubulin-α as positive control) in blocking solution overnight at 4. Next, the membranes were washed and incubated with a horseradish peroxide-conjugated goat anti-mouse secondary antibody diluted in blocking buffer. Proteins were detected by using an enhanced chemiluminescence Western blotting detection kit (Pierce Biotech).

### MTT assay

PANC-1 cells stably transfected with HCCR-1, HCCR-1 siRNA and vector plasmid were plated into 96-well plates in 1 × 10^3 ^cells/100 μl DMEM (Gibco)/well. 20 μl of MTT solution (Sigma) was added into each well daily from the 2nd to 5th day, and plates were incubated for 4 h at 37°C. After removal of the supernatant, 200 ml of dimethyl sulfoxide (DMSO; Sigma) was added to dissolve the crystals. Absorbance values (A) were measured at a wavelength of 490 nm with a microplate reader. Growth curve was made according to the values of 490 nm wavelength light absorption in the three groups The mean ± SD of triplicate assays for each cell line is shown.

### Invasion assay

Matrigel invasion assay was performed by using a 24-well transwell plates(costar) with polycarbonate filters (pore size, 8 μm). The upper side of polycarbonate filter was coated with matrigel (50 μg/ml, BD Biosciences). The chambers were incubated at 37°C with 5% CO2 for 2 h to allow the matrix to form a continuous thin layer. Then the cells stably transfected with HCCR siRNA plasmid and vector plasmid were harvested and 4 × 10^5 ^cells in 200 μl of 0.1% bovine serum albumin were placed in the upper chamber. The lower chamber was filled with 10% serum-medium (700 μl). Cells were cultured for 24 h at 37°C in 5% CO2. Cells on the upper surface of the filter were removed using a cotton swab. Cells invading through the Matrigel and filter to the lower surface were fixed with 4% neutral-buffered formalin and stained with Giemsa. The cell numbers in five fields (up, down, median, left, right. ×200) were counted for each chamber, and the average value was calculated. Assays were done in triplicate for each experiment.

### Luciferase assay

PANC-1 cells stably transfected with vector, constitutively active Akt, or dominant-negative Akt were plated at 1 × 10^5 ^cells in six-well plates and grown for 24 hour before transfection with lipofectime. The cells were co-transfected with three pGL3-Basic vector containing the HCCR-1 proximal promoter regions [pGL3-P1196(+30 to -1166), pGL3-P504 (+30 to -474), pGL3-P423(-52 to -474)] and internal control pRL-CMV using Lipofectamine 2000. The luciferase activity was measured after 24 h of transfection with the luciferase assay kit as indicated by the manufacturer. PRL-CMV was used as an internal standard to normalize the luciferase activity.

### Statistical analysis

All the data were analyzed by SPSS13.0. The positive expression of HCCR in each group was compared using test and ANOVA. The MTT results the data of luciferase assay was analyzed using ANOVA. A test was run for all sites combined and one for each of the site groupings.

## Results

### Antibody preparation of HCCR

**Figure 1:**
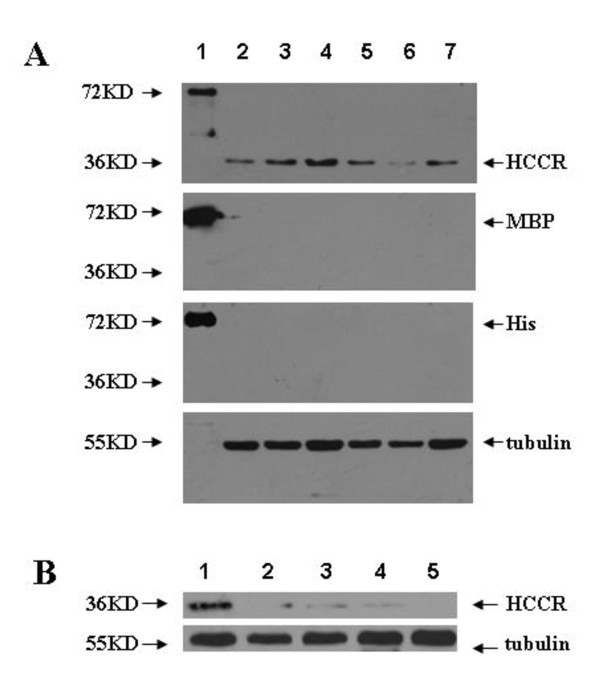
**Analysis of the high specificity of the polyclonal antibody against HCCR protein by Western blotting**. **A) **Lane 1, recombinant pMBP-c-HCCR-His protein (positive control); Lane 2-7, cell lysate from hepatocellular carcinoma tissues. Lane 2-7 showed strong bands by using the polyclonal antibody against HCCR(Mr 36,000) while they showed no bands by using the monoclonal antibody against MBP and His. **B) **Lane 1-5, tissues of hepatocellular carcinoma. Lane 1 showed strong band corresponding to HCCR by using the polyclonal antibody against HCCR. The band was weaken gradually and then disappeared when the antiserum was preabsorbed with gradually increased concentration of recombinant pGEM-HCCR (with a GST tag) protein from Lane 2 to Lane 5.

The polyclonal antibody against HCCR protein, prepared by immunizing Bclb/c mice with the purified recombinant protein pMBPc-HCCR-His, had both high efficiency and specificity which were tested by indirect ELISA (Antibody titer: 1:10000) and Western blot. Western blotting using this polyclonal antiserum showed strong single bands corresponding to HCCR (Mr 36,000) in HCC tissues, which did not react to the monoclonal antibody against MBP and His. Fusion protein pMBPc-HCCR-His served as a positive control (Fig. 1A). The bands corresponding to HCCR (Mr 36,000) disappeared when the antiserum was pre-absorbed with another recombinant protein HCCR-GST (Fig. 1B), suggesting that the polyclonal antibody against HCCR protein had high specificity.

### HCCR-1 overexpression may enhance the pancreatic tumor progression

**Figure 2:**
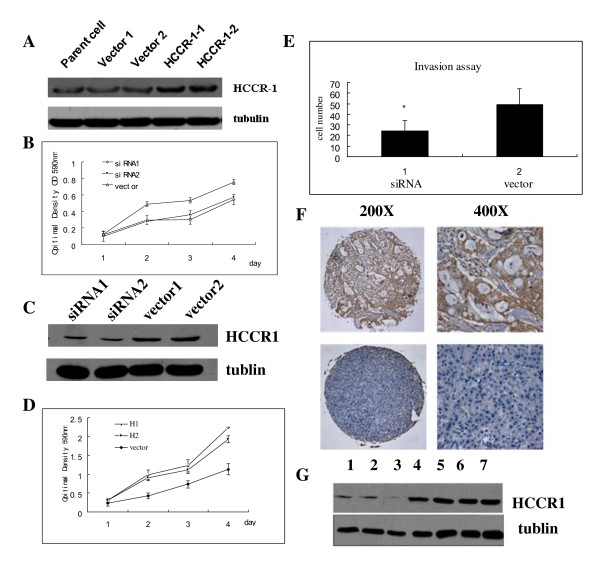
**Overexpression of HCCR-1 induces the proliferation of PANC-1 cells**. **A) **PANC-1 cells were transfected with control empty vector and HCCR-1 gene. **B) **HCCR-1 accelerated the proliferation of PANC-1 cells by 1.5 fold compared to control. The measurement of proliferation rate was performed by MTT assay. Values represent the mean ± SEM, Groups with annotations of H1 and H2 are significantly different with Vector, P < 0.01, two-way ANOVA. **C) **PANC-1 cells were transfected with HCCR-1 siRNA and control empty vector. **D) **Knock-down of HCCR-1 decreased the proliferation of PANC-1 cells MTT assay. Values represent the mean ± SEM, Groups with annotations of siRNA were significantly different with Vector, P < 0.01, two-way ANOVA. **E) **The invasiveness of HCCR-1 siRNA and vector transfectants were assessed by incubating cells in transwell plates(Costar) with polycarbonate filters for 24 h using 10% FBS as a chemoattractant. Cells invaded through the matrigel and filtered to the lower surface were fixed with 4% neutral-buffered formalin and stained with Giemsa. The number of invasion cells was significantly lower in HCCR-1 siRNA transfectants (24.4 ± 9.9) than in vector control(49.1 ± 15.4), *P < 0.01. Each experiment was repeated for three times. **F) **Immunohistochemical staining of pancreatic cancer tissues on a tissue chip. Strong straining of HCCR-1 in a pancreatic carcinoma(the upper two pictures). Expression of HCCR-1 in a paraneoplastic tissue(the two pictures below). The staining was graded according to Allred score system. **G) **The expression of HCCR-1 was analyzed by performing western blotting in the same tissues used in immunohistochemical staining. Lanes 1 to 3, paraneoplastic tissues; lanes4 to 7, pancreatic tumor tissues. Tubulin was used as a loading control.

It has been shown that HCCR-1 is overexpressed in several human cancers [15]. To investigate whether HCCR-1 plays any role in pancreatic cancer development, we firstly examined the pancreatic cancer cell growth in vitro after transfecting PANC-1 cells with HCCR-1 expressing DNA constructs. PANC-1 cells carrying empty vector (pcDNA3.1) were used as controls (Fig. 2A). Our result reveals that HCCR-1 enhances the growth of PANC-1 cells in vitro by 1.5 fold over 3-day incubation period (Fig. 2B). We also obtained the PANC-1 cells which were stably transfected with plasmid containing HCCR siRNA fragment and vector (Fig. 2C). As expected, the proliferation was inhibited in PNAC1 cells stably transfected with HCCR siRNA (Fig. 2D). The growth decreased by 0.65 times and 0.68 times in HCCR-1 siRNA transfected cells. The number of invasion cells was significantly lower in PNAC1 cells transfected with HCCR-1 siRNA (24.4 ± 9.9) than that in vector transfectants (49.1 ± 15.4) (Fig. 2E).

To determine the cellular localization of HCCR-1 protein and its distribution among different tissues, we made the tissue chips implanted with these pathologically different tissues (described in Materials and Method section). HCCR-1 expression was observed in most of pancreatic tumor tissues with the mean Allred score was 4.51 ± 1.549 (Fig. 2F). In contrast, HCCR-1 was expressed at low levels in paraneoplastic tissues and benign tumors and its mean score was 2.87 ± 2.193 (p < 0.001) (Table 1). This result was further supported by the western blot analysis on the same tissues used in the immunohistochemical analyses. As shown in Fig. 2G, strong expression of HCCR-1 was detected in the pancreatic carcinomas compared with which in paraneoplastic tissues. Moreover, it was interesting to note that HCCR-1 expression is associated with histological grade (p = 0.006), the mean score of histological stage II, III (4.53 ± 1.398 and 4.63 ± 1.847) were higher than of histological stage I (3.37 ± 1.461, P < 0.01 respectively), there is no significant difference between stage II and stage III (P = 0.738). Whereas it was not related to clinicopathological factors such as age, sex, nerve infiltration and lymph node metastasis (Table 2). This tissue expression profile suggests that HCCR-1 may function in the pancreatic tumor progression, possibly involving the cellular tumorigenesis signaling pathway.

**Table 1:** HCCR-1 expression in pancreatic cancer tissues, paraneoplastic tissues/benign tumor tissues

	cases	mean score	t	P
Pancreatic cancer	178	4.51	5.853	0.000*
Paraneoplastic tissues/benign tumors	47	2.87		

*P < 0.01

**Table 2:** Relations between HCCR-1 expression and clinicopathological characteristics of tumors

Clinical characteristics	cases	mean score	t	P
Age				

≤60	80	4.64	1.027	0.306
>60	98	4.40		

Sex				

Male	111	4.52	0.187	0.852
Female	67	4.48		

Nerve infiltration				

Yes	99	4.45	0.805	0.42
No	70	4.64		

Lymph node metastasis				

Yes	36	4.69	0.731	0.466
No	134	4.49		

Histological grades			F	

I	19	3.37	5.316	0.006*
II	115	4.53		
III	30	4.63		

*P < 0.01

### HCCR-1 expression is linked to EGF/PI3K/Akt/mTOR signaling in PANC-1 cells

**Figure 3:**
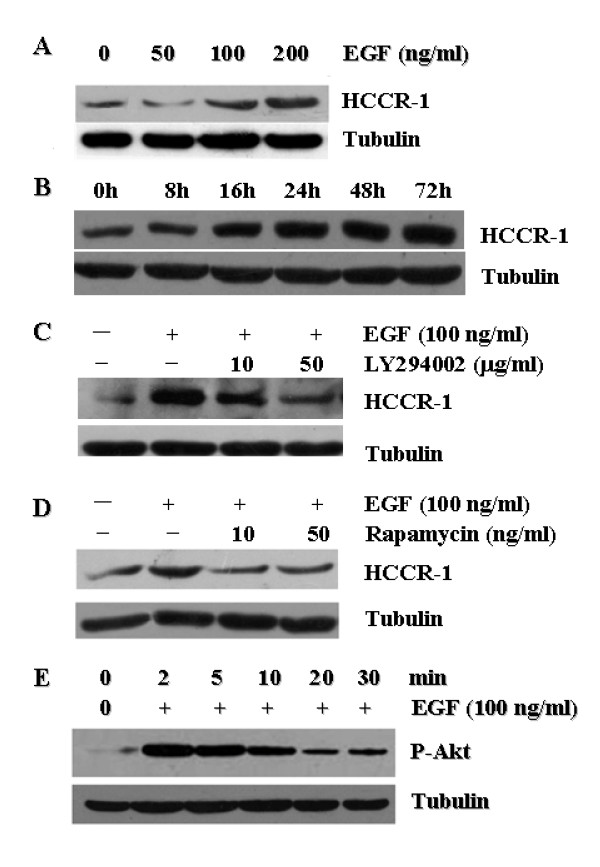
**HCCR-1 overexpression is mediated with EGF-induced PI3K/AKT/mTOR signaling**. Western blot analysis of HCCR-1 expression in PANC-1 cells stimulated with EGF. **A) **Dose dependency (at 24 h). Total protein were extracted from serum starved PANC-1 cells treated with EGF at different concentration, and subjected to Western blotting. **B) **Time course (at 100 ng/ml). Total protein were isolated from PANC-1 cells for Western blotting after different duration of EGF. EGF stimulates HCCR-1 expression in PANC-1 cells in a dose- and time-dependent manner. Tubulin was used as a loading control. Blocking PI3K or mTOR signaling with either **C) **LY294002 (50 μg/mL) or **D) **rapamycin (10 ng/ml), respectively, suppresses the EGF (100 ng/mL)-mediated HCCR-1 expression in PANC-1 cells. **E) **EGF induces the activity of Akt within 2 min at highest level with gradual decrease.

**Figure 4:**
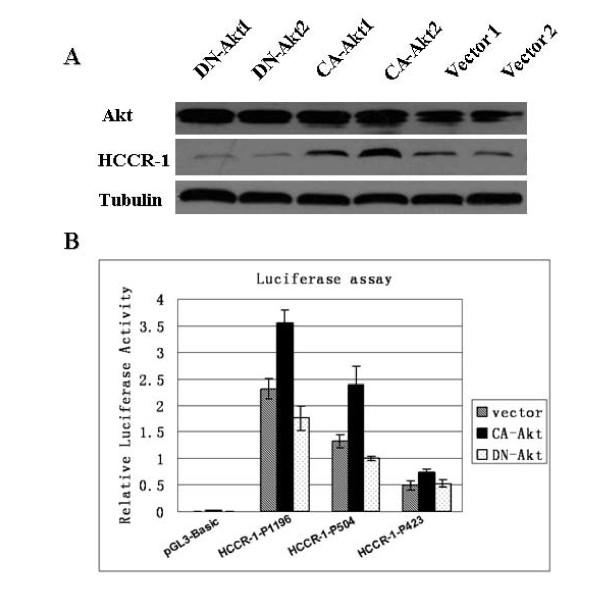
**Akt induces HCCR-1 overexpression by increasing its promoter activity in PANC-1 cells**. **A) **Stable transfectants with two types of constitutively active Akt (CA-Akt1 or 2) induced HCCR-1 expression (lane3,4), whereas dominant negative Akt mutants (DN-Akt1 or 2) failed to induce its expression(lane1,2), compared with vector control(lane5,6). Akt is a doublet protein as shown in the figure. Tubulin, an equivalent loading control of proteins in western blot assays. **B) **Akt transactivates the promoter activity of HCCR-1. The transfectants applied in Fig. 4A were co-transfected with reporter plasmids containing three different constructs of HCCR-1 promoter (for details, see Materials and Methods) and internal control vector. The promoter activity of both pGL3/HCCR-1-P1196 and pGL3/HCCR-1-P504 constructs was enhanced by a constitutively active form of Akt whereas it was down-regulated by a dominant negative mutant form of Akt (P < 0.05, multiple factor ANOVA), and this was not found in the pGL3/HCCR-1-P423 construct. The data are expressed as the mean ± SEM of three separate experiments.

EGF stimulation on PANC-1 cells was commenced to determine whether and how EGF regulates the expression of the HCCR-1 crucial for the pancreatic cancer growth and survival. PANC-1 cells express more HCCR-1 in a dose- and time-dependent manner (Figs. 3A and 3B) when they are stimulated with EGF. The HCCR-1 expression was gradually elevated from 8 h up to 72 h incubation time point, suggesting that EGF signaling is involved in the induction of HCCR-1 expression. The same results were obtained in other two pancreatic cancer cells, SW1990 and CFPAC-1, but the EGF-induced HCCR-1 expression was much significant in PANC-1 cells (data not shown). To further define the HCCR-1 signaling pathway in pancreatic cancer cells, either PI3-kinase inhibitor (LY294002) or mTOR inhibitor (rapamycin) was pre-treated on PANC-1 cells and then they were re-stimulated with EGF. Our result shows that both LY294002 and rapamycin inhibit the up-regulation effect of HCCR-1 by EGF (Figs. 3C and 4D), suggesting that EGF-induced HCCR-1 expression is mediated by PI3K/mTOR signaling in pancreatic cancer. Akt is a key downstream signaling component of the PI3K pathway [27,28], and mediates the downstream effects of mTOR by inducing cell growth, proliferation and survival of malignant cells [29,30]. In cancer cells, Akt is constitutively activated by phosphorylation on residues of serine and threonine at sites 473 and 308, respectively [27]. We examined whether Akt becomes phosphorylated on PANC-1 cells when they are treated with EGF like in other cancer cells. Akt was activated by phosphorylation within 2 min after EGF treatment (Fig. 3E), indicating that Akt serves as a downstream effector of EGF signaling pathway on PANC-1 cells. Taken together, our results suggest that PI3K/mTOR signaling pathway involving Akt plays an essential role for regulating the HCCR-1 levels.

### Akt induces HCCR-1 overexpression by enhancing its promoter activity in PANC-1 cells

In order to gain a better insight into the Akt signaling mechanism on regulating HCCR-1 levels, stable cells lines of PANC-1 cells were established with CA-Akt constructs and DN-Akt mutants. As shown in Fig. 4A, the over-expression of constitutively active form of Akt increased the HCCR-1 levels on stable PANC-1 cell lines whereas dominant negative mutant form of Akt failed to induce HCCR-1 expression as confirmed by western blotting. This result demonstrates that HCCR-1 expression is driven by Akt activity. Previous works have shown that Akt is a key modulator of the HCCR-1 promoter in K562 and NIH/3T3 cells. To test whether Akt regulates the HCCR-1 promoter activity in PANC-1 cells, we generated three reporter constructs containing different proximal promoter regions of HCCR-1 (pGL3/HCCR-1-P1196, pGL3/HCCR-1-P504, and pGL3/HCCR-1-P423). The stable PANC-1 cell lines carrying either CA-Akt or DN-Akt were transfected with reporter constructs and they were assayed for luciferase activity (Fig. 4B). Consistent with the previous work [21], the promoter activity of pGL3/HCCR-1-P423 was the lowest in PANC-1 cells compared to the other two (pGL3/HCCR-1-P1196, pGL3/HCCR-1-P504). However, the promoter activity of pGL3/HCCR-1-P1196 was slightly higher than that of pGL3/HCCR-1-P504 in PANC-1 unlike in K562 and NIH3T3. Interestingly, however, the promoter activity of both pGL3/HCCR-1-P1196 and pGL3/HCCR-1-P504 constructs was enhanced by a constitutively active form of Akt whereas it was down-regulated by a dominant negative mutant form of Akt. This result strongly supports that Akt activity directly regulates the HCCR-1 promoter function. In addition, the Akt-responding element seems to be located in between +30 and -1166 region of HCCR-1 gene. Therefore, Akt seems to be a key regulator of HCCR-1 promoter in pancreatic cancer cells.

## Discussion

Despite of recent advances in understanding the molecular pathogenesis on pancreatic cancer, this disease still remains as one of the most aggressive human solid tumors. The pancreatic cancer is characterized by the rapid growth, metastatic spread, and resistance to chemotherapeutic drugs. This challenging feature of the pancreatic cancer has become the major cause of 40,000 estimated deaths/year in Europe, and nearly 30,000 deaths/year in the USA [31,32].

The accumulated knowledge on the molecular basis of the pancreatic cancers has revealed that many molecular events are responsible for initiating pancreatic cancers and its progression. First, gain or loss mutations in oncogenes or tumor suppressors occur in most of pancreatic cancers (for example, K-ras and p53 mutations found in 75-90% and 50% of pancreatic cancers, respectively) [33]. Secondly, a variety of growth factors and their receptors are expressed at increased levels, such as transforming growth factor-β (TGF-β), epidermal growth factor (EGF), fibroblast growth factor (FGF), and insulin growth factor (IGF), and their receptors. These molecules serve to stimulate the pancreatic cancer cell growth in an autocrine and/or paracrine manner [34,35]. Third, it is also known that molecular aberrations in signaling pathways contribute to the pancreatic cancer. They include Ras-Raf-MEK signaling pathway, the PI3K/Akt signaling pathway, and the signal transducer and activator of transcription (STAT) family of proteins. In addition to molecular lesions described above, there are many other molecular alterations associated with the pancreatic cancer such as mutations affecting p16INKA/retinoblastoma proteins and reactivation of Notch and Hedgehog signaling.

Based on these findings on the molecular alterations of the pancreatic cancers, many therapeutic drugs have been developed by targeting K-ras, EGFR and PI3K/Akt signaling components. Although these therapeutics have promoted the survival of patients, only pancreatic resection improves the survival significantly in patients with advanced pancreatic cancers. Despite of numerous efforts made in exploiting novel targets for pancreatic cancers management, they have been elusive. This means that more extensive studies are needed to understand this disease.

The HCCR-1 gene was first discovered from the cervical cancer in which its expression is elevated [15]. It is over-expressed in many different cancers including leukemia, lymphoma, and carcinomas of the breast, kidney, ovary, stomach, and colon. This suggests that HCCR-1 might provide the fundamental function essential for tumor growth and survival. Indeed, HCCR-1 was capable of transforming, almost as efficient as Ras, the NIH/3T3 and Rat1 cells. It also could impart those cells the ability to form the tumor in vivo. The underlying mechanism on the HCCR-1-mediated tumorigenesis was p53 stabilization concomitant with suppression of p21 and Bax, and the interaction with its binding proteins [15]. The subsequent studies on HCCR-1 revealed that it also exerts an oncogenic activity in the breast and colon cancers [16,20]. It was the PI3K/Akt pathway that governs the expression of HCCR-1 in cancer cell lines such as K562, MCF7, and A549 [21]. As explained above, PI3K/Akt pathway is one of the downstream signaling pathways triggered by EGF-EGFR ligation, and it regulates cell survival, proliferation, and resistance to apoptosis.

Previous studies suggested that the COOH-terminal sequence YLGTRR appears to be a major linear epitope of HCCR [18]. In the present study, we successfully developed a highly efficient and specific polyclonal antibody against HCCR by cloning the cDNA encoding a polypeptide homologus to the 167th - 360th amino acid residues of HCCR into the pMBP vector. Using the polyclonal antibody, we found that HCCR-1 is over-expressed in most of pancreatic tumors and its expression level is associated with the progression of the disease (Fig. 1C). On the contrary, it is expressed less and at low levels in paraneoplastic tissues and benign tumors (p < 0.01) (Table 1). This is consistent with our idea that HCCR-1 function is required for the pancreatic cancer progression.

Interestingly, the over-expression of HCCR-1 found in most of pancreatic cancers was triggered by EGF signaling which has been already known to regulate the pancreatic cancer development. Upon EGF stimulation, EGFR initiates the activation of proliferative and survival signaling pathways, such as the Ras/Raf/MEK (MAPK) and Akt/mTOR cascades [36]. Our study clearly shows that EGF-induced over-expression of HCCR-1 is mediated by the PI3K/Akt/mTOR signaling pathway. It suggests that HCCR-1 is one of the down-stream components of the EGF-triggered PI3K/Akt/mTOR signaling which plays a pivotal role in the pancreatic cancer tumorigenesis. During this process, activated Akt directly modulated the promoter activity of HCCR-1 located in the 5'upstream region of HCCR-1 gene. Since the -1166 to +30 region of HCCR-1 gene contains many other putative binding motifs for other transcription factors such as E2F, GATA-1, and estrogen (estrogen-related) [21], further investigation is needed to identify other potential mediators regulating the HCCR-1 expressions in the pancreatic cancers.

## Conclusions

In conclusion, although further studies are required to fully address the molecular mechanism of HCCR-1 on the pancreatic tumorigenesis, our result provides the insight on the role of HCCR-1 and its involvement in the pancreatic cancer via the EGF-triggered PI3K/Akt/mTOR pathway. Therefore, our work suggests that HCCR-1 could be a potential target for pancreatic cancer therapeutics.

## Competing interests

The authors declare that they have no competing interests.

## Authors' contributions

ZX and YZ participated in the design of the study and performed the statistical analysis. YZ and JJ carried out the molecular genetic studies and drafted the manuscript. YY, RS, BH, ZZ and ZH conceived of the study, and participated in its design and coordination. GZ and JWK participated in the design of the study. All authors read and approved the final manuscript.

## Pre-publication history

The pre-publication history for this paper can be accessed here:

http://www.biomedcentral.com/1471-2407/10/161/prepub
